# 

**Published:** 1888-02

**Authors:** 


### SOCIETY NOTES.

### AUSTIN DISTRICT MEDICAL SOCIETY. THIRD QUARTERLY MEETING.

AN INVITATION TO THE PROFESSION.

AUSTIN, TEXAS, FEBRUARY 8, 1888.

DEAR DOCTOR:—It affords us pleasure to announce the third quarterly meeting of the Austin District Medical Society, which will be held in the city of Austin on the 8th of March, *prox.* It is gratifying to state that the prospects for a good meeting are flattering. A number of interesting papers will be read and discussed, prominent among which will be an article by Dr. R. S. Gregg, of Manor, on the important subject of the "Treatment of Abortions"—discussion will be opened by Dr. Sam. Cunningham, of Elgin. One by Dr. F. R. Martin, of Kyle, on "Pneumonia"—discussion opened by Dr. R. M. Swearingen. One by Dr. G. B. Underhill, of New Braunfels—subject not announced—discussion opened by Dr. J. W. McLaughlin. Dr. F. E. Daniel has promised a paper on some subject in Dermatology.

In addition to the above, Dr. A. N. Denton will read a paper on the "Management and Treatment of the Insane in Texas." Dr. L. W. Cock, of San Marcos, will also entertain the Society with an essay entitled "Is the World Satisfied with the Medical Profession." Dr. Wooten will discuss the question.

You are respectfully invited to be present and participate in the discussions. Voluntary papers and reports of cases are always in order.

The place of meeting is over Tobin's drug store, in the Hall of Travis County Medical Society. Time, 10 o'clock a. m. There will be an afternoon and a night session.

Hoping to have the pleasure of seeing you and having your valuable experience and encouragement at this meeting, we are,

Very respeetfully,

T. J. BENNETT, Secretary.

THOS. D. WOOTEN, President.

